# ﻿*Eumachiabrevipedunculata* (Rubiaceae, Palicoureeae), a new species from Yunnan, China

**DOI:** 10.3897/phytokeys.255.143380

**Published:** 2025-04-25

**Authors:** Dong-Li Quan, Yun-Hong Tan

**Affiliations:** 1 Southeast Asia Biodiversity Research Institute & Center for Integrative Conservation, Xishuangbanna Tropical Botanical Garden, Chinese Academy of Sciences, Mengla, 666303, Yunnan, China Xishuangbanna Tropical Botanical Garden, Chinese Academy of Sciences Mengla China; 2 Yunnan International Joint Laboratory of Southeast Asia Biodiversity Conservation & Yunnan Key Laboratory for Conservation of Tropical Rainforests and Asian Elephants, Menglun, Mengla, 666303, Yunnan, China Yunnan International Joint Laboratory of Southeast Asia Biodiversity Conservation & Yunnan Key Laboratory for Conservation of Tropical Rainforests and Asian Elephants Mengla China

**Keywords:** Flora, Indo-China, new taxon, *Psychotria*, Psychotrieae

## Abstract

*Eumachiabrevipedunculata* is a newly-identified species of the tribe Palicoureeae of Rubiaceae from Yunnan, China. It is morphologically similar to *E.straminea*, but differs by its smaller stipules, shorter petioles, smaller leaf blades, reduced inflorescences with shorter peduncles, axes and pedicels, longer calyx lobes, yellow-green flowers with shorter corolla tubes and shorter anthers. This species is widely found in southern Yunnan, but has long been misidentified as *Psychotria*. We clarify its taxonomic status and provide a description and illustration here.

## ﻿Introduction

The pantropical genus *Eumachia* DC. (Rubiaceae, Palicoureeae) is characterised by the following characteristics: raphides in the tissues; shrub or small tree habit; smooth branchlets; flattened young shoots and young internodes; opposite leaves, drying yellowish-green; interpetiolar stipules that are persistent or fall by fragmentation, usually united around the stem, generally glandular when young, but becoming indurated and yellowed to ochre when old; terminal inflorescences with green to white axes and typically short bracts; white to cream or yellowish-green corollas with valvate aestivation; usually barbate throats; pyrenes adaxially plane to concave, with marginal preformed germination slits and lacking ethanol-soluble pigments; non-ruminate endosperm (Andersson 2001; Taylor 2005; Barrabé et al. 2012; Taylor et al. 2017). In contrast, its close relative *Psychotria* L. features vegetative parts drying reddish-brown to grey, deciduous stipules and pyrenes containing ethanol-soluble pigment (Andersson 2001; Davis et al. 2001; Barrabé et al. 2012; Taylor et al. 2017; Razafimandimbison and Rydin 2024).

The genus *Eumachia* was established in 1830, with the type species being *E.carnea* (G.Forst) DC. (de Candolle 1830). Since then, the genus has been treated as a synonym of *Psychotria* (Robbrecht 1988, 1993), though the characteristics of *E.carnea* do not match the latter. More recently, studies based on molecular phylogeny and morphology resurrected the genus *Eumachia* and transferred it from the tribe Psychotrieae to Palicoureeae (Barrabé et al. 2012; Barrabé and Davis 2013; Applequist 2014; Razafimandimbison et al. 2014). Some comprehensive combinations and treatments were subsequently made, expanding the circumscription of *Eumachia* (Barrabé et al. 2013; Delprete and Kirkbride 2015; Taylor et al. 2017). So far, 86 species have been reported in the genus, ranging from Palaeotropical to Neotropical (POWO 2025). Only four species are known from continental Asia (Taylor et al. 2017; Turner 2018). Two species *E.montana* (Blume) I.M.Turner and *E.straminea* (Hutch.) Barrabé are currently known in China (Wilson 1916; Chen and Taylor 2011; Taylor et al. 2017; Bijmoer et al. 2024).

Through our study of *Psychotria* in China, we identified a species extensively collected across southern Yunnan and Laos. However, it does not correspond to any previously recognised taxa. A thorough morphological analysis and consultations of relevant literature and specimens indicate that this species shares characteristics with *Eumachia* and is new to science. While it is similar to *E.straminea* in features such as its glabrous stems, papery (when dried) and elliptic-lanceolate leaf blades with 6–8 pairs of secondary veins and terminal inflorescences, it can be distinguished by its smaller stipules, leaf blades and inflorescences and by differences in the floral parts, as outlined in the conclusions below.

We described and illustrated the new *Eumachia* species here, including formal nomenclatural and morphological details, an explanation of the epithet, selected photographs, an overview of its habitat and phenology and a comparison with similar species that may be confused. Additionally, we included data on studied specimens, a preliminary conservation assessment following the IUCN Red List Categories and Criteria (IUCN 2012; IUCN Standards and Petitions Committee 2024) and notes on its distinctive morphological characteristics and ecological traits.

## ﻿Materials and methods

The materials studied were derived from living plants collected in the field and herbarium specimens deposited at Xishuangbanna Tropical Botanical Garden (HITBC). To identify the morphological differences with related species, we examined specimens from the Herbaria K, MO, NY, P, IBK, IBSC, LE, A, E etc., through online databases including JSTOR Global Plants (https://plants.jstor.org/), CVH (https://www.cvh.ac.cn/), NSII (http://www.nsii.org.cn/) and GBIF (https://www.gbif.org/). Descriptions were based on morphological observations and measurements of living plants and herbarium specimens.

## ﻿Taxonomy

### ﻿ Eumachia brevipedunculata

Taxon classificationPlantaeGentianalesRubiaceae

Y.H.Tan & D.L.Quan sp. nov.

95C4A7A6-1571-5E1D-8F36-ECC53A6B99F2

urn:lsid:ipni.org:names:77360587-1

Fig. 1

#### Type.

China • Yunnan Province: Xishuangbanna Dai Autonomous Prefecture, Mengla County, Menglun Township, Xishuangbanna Tropical Botanical Garden, tropical rain forest, 21°54′52.51″N, 101°15′25.92″E, 579 m a.s.l., 13 May 2024, flowering, *D.L.Quan & Y.H.Tan TYH3288* (holotype, HITBC0115875!; isotypes, HITBC0115876!, HITBC0115877!).

**Figure 1.**
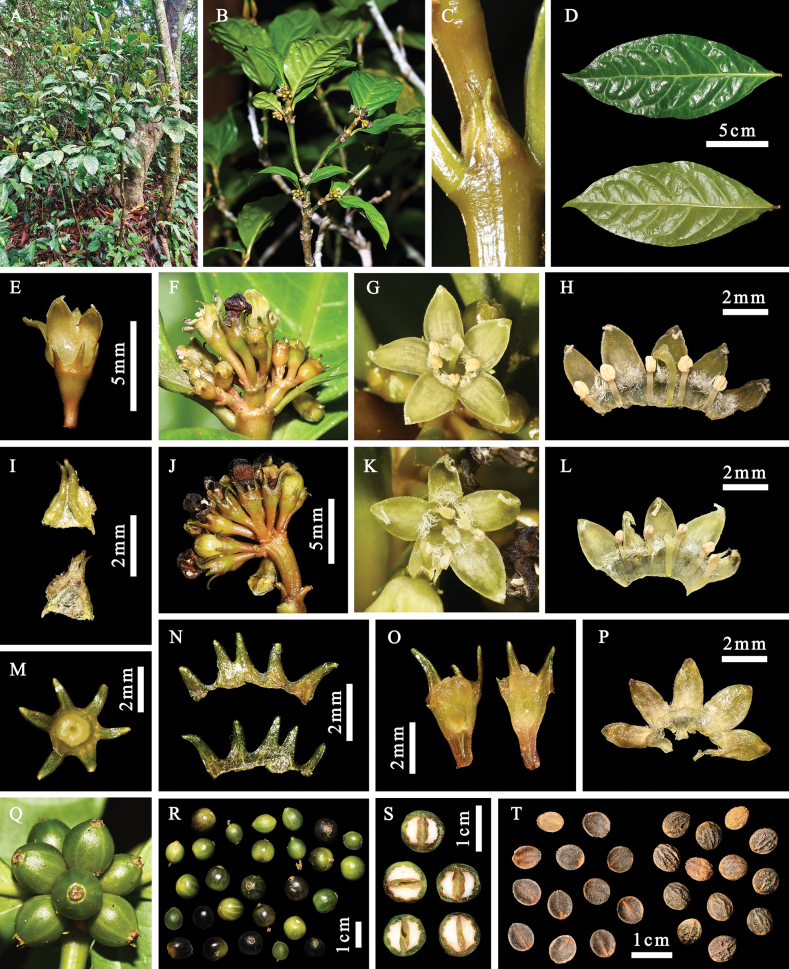
*Eumachiabrevipedunculata* Y.H.Tan & D.L.Quan **A** habitat **B** habit **C** stipules **D** leaves **E** flower showing calyx, hypanthium and corolla **F** inflorescence **G** front view of long-styled flower **H** interior view of longitudinally opened long-styled flower **I** abaxial and adaxial surface of stipule **J** inflorescence **K** front view of short-styled flower **L** interior view of longitudinally opened short-styled flower **M** front view of calyx lobes and disc **N** abaxial and adaxial view of longitudinally opened calyx **O** longitudinal section through hypanthium, disc and calyx **P** abaxial view of opened corolla **Q** infructescence **R** fruits at different developmental stages **S** cross-section of fruits, showing pyrenes and seeds **T** ventral (left) and dorsal (right) view of pyrenes. Photographs by Dong-Li Quan.

#### Diagnosis.

*Eumachiabrevipedunculata* is morphologically similar to *E.straminea*, but can be distinguished by its smaller stipules (ca. 2 mm vs. 2.5–6 mm long), shorter petioles (0.5–1.2 cm vs. 1–2 cm long), smaller leaf blades (5–18 × 2–8 cm vs. 10–25 × 4–10 cm); smaller inflorescences (1–1.2 × 1–1.2 cm vs. 1–4 × 1–2.5 cm) without developed axes (vs. axes 3–10 mm long), shorter peduncles (up to 0.5 cm vs. 1–1.5 cm long), shorter pedicels (up to 1.5 mm vs. 1.5–4 mm long); longer calyx lobes (0.5–1.5 mm vs. up to 0.5 mm long), linear-lanceolate to narrowly ligulate calyx lobes (vs. denticulate), shorter corolla tubes (1–1.5 mm vs. 1.5–2 mm long), yellowish-green corolla (vs. white to cream) and shorter anthers (ca. 0.5 mm vs. ca. 1 mm long).

#### Description.

Shrubs, 0.5–2.5 m tall, branched; ***stems*** terete, glabrous; ***internodes*** flattened. ***Leaves*** opposite, without domatia; ***petiole*** 0.5–1.2 cm long, glabrous; ***leaf blade*** concavo-convex, elliptic to lanceolate, 5–18 × 2–8 cm, green, often paler abaxially, adaxially somewhat shiny in life, drying papery, glabrous on both surfaces, base cuneate to attenuate, apex acute to acuminate, margins flat or slightly undulant, usually thinly revolute; ***secondary veins*** 6–8 at each side of the mid-rib, free or forming a weakly- to a well-developed looping submarginal vein, adaxially costa thickened to prominent and secondary veins sometimes prominent, abaxially costa and secondary veins prominent, remaining venation flat; ***stipules*** persistent or falling by fragmentation with persistent portion becoming indurated and yellow to ochre, interpetiolar to shortly fused around stem or forming a sheath, ca. 2 mm long, triangular to ovate, with 2 costae bearing 2 lobes, lobed to 1/4–1/2, lobes subulate with glandular tip, abaxially glabrous, adaxially at base with well-developed drying red-brown colleters. ***Inflorescences*** terminal, cymose, congested, subglobose to corymbiform, 1–1.2 × 1–1.2 cm, branched to 1–3 orders without developed secondary axes, generally 3- to 15-flowered, glabrous, subsessile to shortly pedunculate with ***peduncle*** up to 5 mm long; ***bracts*** narrowly triangular, ca. 0.5 mm long, pubescent to glabrescent outside, apex obtuse; ***pedicels*** up to 1.5 mm long. ***Flowers*** 5-merous, rarely 4- or 6-merous, usually bent down, distylous; **hypanthium** obconic, ca. 1.5 mm long, glabrous; ***calyx*** green to brown, glabrous, limb ca. 0.5 mm long, lobes 0.5–1.5 mm long, lobes linear-lanceolate to narrowly ligulate, apex obtuse; ***corolla*** yellowish-green, campanulate, tube 1.5–2.5 mm in diameter, 1–1.5 mm long and slightly shorter than half the corolla length, glabrous outside, inside white villous in throat, lobes valvate in bud, ovate, 2–2.5 × 1–1.5 mm, adaxially rostrate, abaxially smooth, apex obtuse, bent inwardly; ***stamens*** glabrous, in long-styled form 1.5–2 mm long, slightly exserted from corolla tube, in short-styled form 2.5–3 mm long, completely exserted from corolla tube; ***anthers*** elliptic to oblong, obtuse, dorsifixed, ca. 0.5 mm long, always exserted from corolla tube; ***filaments*** in long-styled form 1–1.5 mm long, in short-styled form 2–2.5 mm long; ***stigmas*** 2-lobed, lobes ca. 0.2 mm long; ***style*** erect, clavate; style and stigma in long-styled form 2–2.5 mm long, can exceed 1/2 of the corolla tube, but is never exserted from the corolla tube, in short-styled form 1.5–2 mm long, equal in length to or partially exserted from corolla tube; ***ovary*** inferior, 2-celled, ovules 1 in each cell, disc glabrous. ***Fruit*** drupaceous, fleshy, ellipsoid to subglobose, 0.6–1 cm in diameter, stipitate, glabrous, black at maturity, not reddening, ribbed when dried, calyx lobes persistent; ***pyrenes*** 2, hemispherical, bony, dorsally convex with granulose ornamentation or 1–5 shallowly ribbed, ventrally plano-concave, with marginal preformed germination slits, without ethanol-soluble pigments; ***seeds*** ellipsoid to hemispherical, 4.5–6 × 5–7 mm; ***endosperm*** fleshy, non-ruminate.

#### Phenology.

Flowering from April to July, fruiting from June to April.

#### Etymology.

The specific epithet ‘*brevipedunculata*’ refers to the new species’ distinguishing feature of reduced inflorescences with short peduncles, axes and pedicels. Its Chinese name is given as 短序肉沛木 (Pinyin: duǎn xù ròu pèi mù).

#### Habitat and distribution.

**Figure 2.**
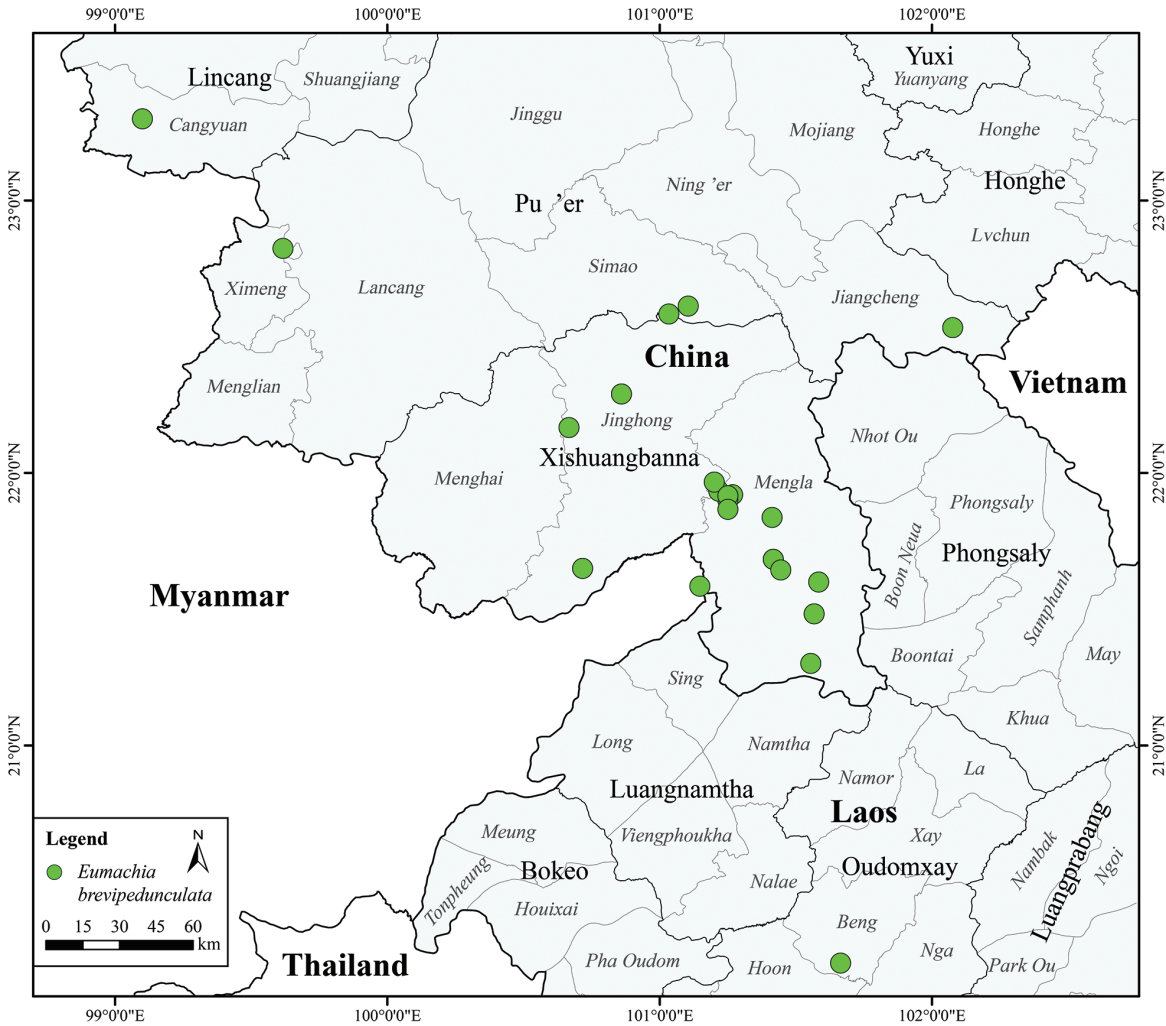
Distribution of *Eumachiabrevipedunculata* Y.H.Tan & D.L.Quan.

This species thrives in tropical rainforests and humid evergreen broadleaf forests at 500–1200 m a.s.l. It has been collected from 23 locations in Yunnan and one site in Laos (Fig. 2).

#### Preliminary IUCN Conservation status.

According to the current distribution of *Eumachiabrevipedunculata*, the species is found in 23 locations in Yunnan and Laos. *Eumachiabrevipedunculata* relies heavily on the formal conservation of primary habitat. More than 10 locations are situated within well-protected nature reserves or rainforests. However, some populations are located at the edge of these reserves or near roads, making them vulnerable to threats such as vegetation clearance, destruction by livestock or construction activities. The subpopulations are dispersed and relatively small in size and area, with an estimated total population of about 4,500 individuals. The extent of occurrence (EOO) for *E.brevipedunculata* is 41,638 km^2^ and the area of occupancy (AOO) is 88 km^2^, as calculated by ShinyGeoCAT (Moat et al. 2023). Based on the IUCN Red List Categories and Criteria (IUCN 2012), notably on the number of locations and the EOO, this species qualifies for classification as Least Concern (LC).

#### Notes.

*Eumachiabrevipedunculata* features persistent or marcescent stipules, preformed germination slits, lacks ethanol-soluble pigments in the pyrenes and has a yellowish-green drying colour, which are general characteristics of the tribe Palicoureeae. In *Eumachia*, fruits are typically orange to red when ripe; however, the fruits of *E.brevipedunculata* and *E.straminea* are black. However, colourful fruits, for example, red, blue, white or black, are known to occur in Palicoureeae. *Eumachia* is not easy to diagnose morphologically because some characteristics were found to be widely variable, such as the stipule type (fused into a tube), the stem texture (smooth or corky), the structure of the inflorescences (lax to subcapitate), the shape of the pyrenes (abaxially smooth to ribbed and adaxially plano-concave to having one or two longitudinal grooves), the type of endosperm (entire to variously ruminate) (Andersson 2001, 2002; Razafimandimbison et al. 2014; Taylor et al. 2017; Taylor 2020; Santos et al. 2021). Moreover, the yellowish-green drying colour of the vegetative parts is generally regarded as a typical and diagnostic characteristic of *Eumachia* contrasted with reddish-brown to grey in *Psychotria* (Barrabé et al. 2012; Taylor et al. 2017). However, some studies considered that the colour of the dried leaves varies considerably with how the specimen was prepared and conserved (Taylor 2020; Taylor et al. 2023). All these variations increase the difficulty of intergeneric and interspecific identification of *Eumachia*. While these characteristics may be homoplasious and not necessarily diagnostic, they remain taxonomically useful (Taylor 2020). Additional taxonomically informative features of *Eumachia* include chemical constituents, the development of tertiary and quaternary leaf venation, leaf anatomy and the morphology of domatia and stomata (Taylor 2016; Berger et al. 2022).

**Figure 3.**
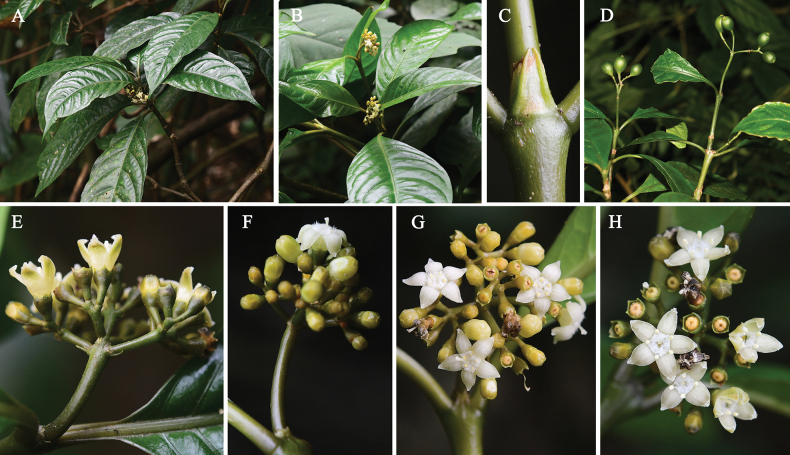
*Eumachiastraminea* (Hutch.) Barrabé, C.M.Taylor & Razafim **A, B** habit **C** stipule **D** infructescence **E** inflorescence **F** inflorescence of long-styled flowers in lateral view **G** inflorescence of long-styled flowers in front view **H** inflorescence of short-styled flowers in front view. Photographs by Xiao-Wen Liao (**A–C, E–H**) and Xiao-Dong Zeng (**D**).

*Eumachiabrevipedunculata* is widely distributed in southern Yunnan, China. It has long been misidentified as a *Psychotria* species, for example, as *P.straminea* (synonym of *E.straminea*), *P.henryi* H.Lév., *P.asiatica* L., *P.siamica* Hutch. and so on. It is most similar to *E.straminea* (Fig. 3), but differs by its smaller stipules and leaf blades, shorter petioles, reduced inflorescences and differences in the floral parts such as longer calyx lobes, shorter corolla tubes and anthers and yellow-green (vs. white-cream) flowers (Table 1). The new *Eumachia* species is often confused with *P.henryi* because of the terminal inflorescences, the subcapitate or congested cymose and subsessile inflorescences, but it is distinct by the broader and glabrous leaf blades, the abaxially glabrous, persistent stipules, the undeveloped inflorescence axes, the smaller bracts, the glabrous calyx, the yellowish-green and campanulate corolla, the ovate and larger corolla lobes and the green-black and smaller fruits.

**Table 1.** Morphological comparison of *Eumachiabrevipedunculata* and *E.straminea* (characters of *E.straminea* from Wilson (1916) and Chen and Taylor (2011) and the examined type specimens MO2536011 and MO2536013).

Characters	*E.brevipedunculata*	*E.straminea*
Stipule length	2 mm	2.5–6 mm
Petiole length	0.5–1.2 cm	1–2 cm
Leaf size	5–18 × 2–8 cm	10–25 × 4–10 cm
Inflorescence size	1–1.2 cm in diameter	1–4 × 1–2.5 cm
Length of inflorescence axes	ca. 0–0.3 cm	0.3–1 cm
Peduncle length	up to 5 mm	1–1.5 cm
Pedicel length	up to 1.5 mm	1.5–4 mm
Calyx lobe length	0.5–1.5 mm	up to 0.5 mm
Calyx lobe shape	linear-lanceolate to narrowly ligulate, apex obtuse	denticulate
Corolla colour	yellowish-green	white to cream
Corolla tube length	1–1.5 mm	1.5–2 mm
Anther length	ca. 0.5 mm	ca. 1 mm

#### Additional specimens examined (paratypes).

China • Yunnan Province: Xishuangbanna Dai Autonomous Prefecture: Mengla County: rainforest, 21°29′N, 101°34′E, 800 m a.s.l., 29 May 1982, flowering, *expedition team 32655* (HITBC0002822) • Mengla Township: Bubeng Village, 21°36′N, 101°35′E, 720 m a.s.l., 27 April 1982, flowering, *expedition team 31873* (HITBC0002825) • Menglun Township: forestry farm, 21°55′N, 101°15′E, 12 May 1975, flowering, *P.Z.Zhu 12612* (HITBC0002833) • in the Menglun sub-reserve of Xishuangbanna National Nature Reserve, 21°56′N, 101°13′E, 650 m a.s.l., 11 August 1975, fruiting, *G.D.Tao 13730* (HITBC0002839) • Xiaola highway 53 km, dry open forest on the mountaintop, 21°58′N, 101°12′E, 900 m a.s.l., 22 October 1973, fruiting, *P.Z.Zhu 10402* (HITBC0002834) • Qixiang, dry forest, 21°55′N, 101°15′E, 1200 m a.s.l., 23 May 1961, flowering, *Y.H.Li 3278* (HITBC0002828) • Chengzi Village, 21°52′N, 101°15′E, 20 September 1972, fruiting, *G.D.Tao 7035* (HITBC037229, HITBC0002836) • Mengxing Village, ravine rainforest over limestone, 21°50′12.45″N, 101°24′45.25″E, 609 m a.s.l., 16 May 2021, flowering, *J.W.Li 6810* (HITBC0075681) • Xishuangbanna Tropical Botanical Garden: 21°54′52.69″N, 101°15′24.30″E, 576 m a.s.l., 24 January 2024, fruiting, *D.L.Quan & Y.H.Tan TYH3271* (HITBC0121858) • 21°55′11.86″N, 101°16′4.19″E, 553 m a.s.l., 28 April 2024, flowering, *D.L.Quan & Y.H.Tan TYH3280* (HITBC0121859) • 21°55′10.63″N, 101°16′7.14″E, 561 m a.s.l., 30 November 2024, fruiting, *D.L.Quan TYH3299* (HITBC0121860) • 21°54′N, 101°15′E, 540 m a.s.l., 2 January 1959, fruiting, *Y.H.Li 213* (HITBC0002841) • 21°54′N, 101°15′E, 570 m a.s.l., 13 July 1959, fruiting, *Y.H.Li 1602* (HITBC0002840) • 21°41′N, 101°25′E, 570 m a.s.l., 27 April 2011, flowering, *J.X.Hu C420152* (HITBC0022962) • 21°41′N, 101°25′E, 570 m a.s.l., 28 April 2011, fruiting, *J.X.Hu C420196* (HITBC0022961) • 21°41′N, 101°25′E, 570 m a.s.l., 24 June 2009, fruiting, *W.Q.Xiao C400518* (HITBC0022957, HITBC0034323, HITBC0034324) • Guanlei Township, near border, 21°35′5.88″N, 101°8′51.30″E, 596 m a.s.l., 29 April 2021, flowering, *J.W.Li 6636* (HITBC0075499) • on the way from Mengyuan Village to Longlin Village, 21°38′37.89″N, 101°26′39.59″E, 1005 m a.s.l., 16 April 2021, flowering, *S.K.Peng P1341* (HITBC0076112) • Shangyong Township, Longmen Village, 21°18′4.84″N, 101°33′16.03″E, 971 m a.s.l., 10 May 2019, flowering, *S.S.Zhou, J.H.Li & L.X.Wang G4-285* (HITBCXSBN002713) • Yiwu Township, beside the river, 1000 m a.s.l., 9 December 2010, fruiting, *J.T.Yin 1879* (HITBC0030358) • Jinghong: Nabanhe, beside the river, 22°10′N, 100°40′E, 700 m a.s.l., 7 November 1988, fruiting, *G.D.Tao 44896* (HITBC0002835) • Dadugang Township, Dahuangba Village, tropical lowland rainforest, 22°17′24.64″N, 100°51′34.40″E, 995 m a.s.l., 7 July 2019, flowering, *J.W.Li 4857* (HITBC0035650) • Menglong Township: Manyangguang Village, 21°39′N, 100°43′E, 16 October 1978, fruiting, *G.D.Tao 19704* (HITBC037215, HITBC037221, HITBC037231, HITBC0002838) • Guanglong Mountain, 21°39′N, 100°43′E, 800 m a.s.l., 6 November 1958, fruiting, *S.W.Zhao 0169* (HITBC0002814) • Mengyang Township, in the Mengyang sub-reserve of Xishuangbanna National Nature Reserve, 22°17′25″N, 100°51′35″E, 992 m a.s.l., 7 July 2019, flowering, *J.W.Li & L.Wang G3-788* (HITBC-XSBN001702) • Puwen, Dakaihe, 22°35′N, 101°2′E, 840 m a.s.l., 14 August 1977, fruiting, *G.D.Tao 16937* (HITBC0002837) • Lincang: Cangyuan County: Banhong Township, humid ravine rainforest, 23°18′N, 99°6′E, 750 m a.s.l., 19 May 1974, flowering, *Y.H.Li 11528* (HITBC0002827) • Pu ‘er City: Jiangcheng County: ravine rainforest beside the Tuka River, 4 August 2011, fruiting, *S.S.Zhou 10482* (HITBC0002897) • Simao District: Nanping Township, Taiyanghe Provincial Nature Reserve, forest by the ravine, 1 September 2011, fruiting, *S.S.Zhou 10760* (HITBC0002787) Ximeng County: Zhongke Township, near river, 22°49′28.47″N, 99°36′58.35″E, 854 m a.s.l., 26 June 2020, flowering, *D.P.Ye 1067* (HITBC0062765, HITBC0062766). Laos • Oudomxay Province: Beng Township: Nahom Village, sandy soil wet valley rain forest, 880 m a.s.l., 3 November 1996, flowering, *H.Wang 2392* (HITBC0002829).

## Supplementary Material

XML Treatment for Eumachia brevipedunculata

## ﻿Additional information

### Author ORCIDs

Dong-Li Quan https://orcid.org/0000-0002-0749-2007

Yun-Hong Tan https://orcid.org/0000-0001-6238-2743

### Data availability

All of the data that support the findings of this study are available in the main text.
